# Pattern of Skin Diseases at University of Benin Teaching Hospital, Benin City, Edo State, South-South Nigeria: A 12 Month Prospective Study

**DOI:** 10.5539/gjhs.v4n3p148

**Published:** 2012-05-01

**Authors:** B. A. Ukonu, E. U Eze

**Affiliations:** 1 University of Abuja Teaching Hospital, Gwagwalada, Abuja, Nigeria; 2 University of Benin Teaching Hospital, Benin City, Edo State, Nigeria

**Keywords:** skin diseases, eczematous dermatitis, infectious skin diseases

## Abstract

**Background and Objective:**

This study aims to look at the pattern and incidence of skin diseases seen in Dermatology/Venereology clinic at the University of Benin Teaching Hospital, Benin City, Edo State, South-South Zone, Nigeria and compare it with other zones of Nigeria.

**Materials and Methods:**

This was a prospective study on pattern and incidence of skin diseases in new patients presenting at the Dermatology/Venereology outpatient clinic of the University of Benin Teaching Hospital, Benin City, Edo State, South-South, Nigeria, from September 2006 to August 2007. All patients were seen by the researchers. Diagnosis were made clinically and sometimes with the support of histopathology.

**Results:**

A total number of 4786 patients were seen during the study period and these comprised 2647 HIV/AIDS patients and 2112 pure Dermatological patients. Out of 4786 patients, 755 (15.8%) were new patients. The new patients comprised 96 (12.7%) children patients (< 15 years) and 659 (83.7%) adult patients (>15years). The ages of the patients ranged from 2 weeks to 80 years and more than two-third were < 40 years. There were 354 males (46.9%) and 401 females (53.1%). This represents female: male ratio of 1.1: 1. Eczematous dermatitis accounted for 20.9% of the skin diseases and was the most common of the skin diseases observed. This is consistent with observation from other zones in Nigeria. Other skin diseases observed in order of frequencies include: Papulosqamous disorder (9.0%), Infectious skin diseases like fungal, viral, bacterial and parasitic infestation, at 7.9%, 7.7%, 2.3% and 2.1% respectively. Pigmentary disorders (5.0%), hair disorders (4.2%) and Benign neoplastic skin disease (6.5%). All the patients that had neurofibromatosis were females (1.9%). HIV-related skin diseases were observed to have increased remarkably (7.9%) with Kaposi’s sarcoma, papular pruritic eruptions and drug eruptions being the commonest mode of presentation.

**Conclusion:**

The current pattern of skin diseases in Benin City, South-South Nigeria seems to follow a similar pattern observed in other Geo-political zones in Nigeria. The eczematous dermatitis took the lead and the impact of HIV-related skin diseases were vividly noticed to be on the increase. Connective tissue disorder and cutaneous malignancies were low in their occurrences. Our findings showed no major differences in the pattern of skin diseases when compared with other zones of Nigeria. Allergic skin diseases were observed to be on the increase in all the geo-political zones; possibly due to increase in urbanization and its attending socio-economic burden.

## 1. Introduction

The skin is a treasury of important lesions that can usually be recognized clinically. In primary care, skin problems are becoming more important and the prevalence of some common skin conditions, such as atopic dermatitis and skin cancer are on the increase (Julian, 1999). Currently skin disorders account for about 15% of all consultations in general practice in the United Kingdom (Keith, 1984), but this does not show a true reflection of skin disease burden since majority of patients end-up ignoring their symptoms or going to quacks in their community (Keith, 1984).

In a household survey of two villages in north-western Tanzania, significant skin disease was found in 27% of the individuals examined (Gibbs, 1996). Increased urbanization has also been noticed to influence the trend of skin diseases, especially areas that are not properly planned and have little or no social amenities. A study of non-specialized health centres in Bamako, Mali, found that up to 26% of consultations were due to skin conditions in some centres (Mahe N’Diaye & Bobin, 1997).

Interestingly, most of the skin diseases are preventable, treatable and rarely fatal (Mallon, Newton, Klassin, et al. 1999) although its effect on quality of life can be intriguingly undesirable. Therefore, it is important that the knowledge of the incidence and pattern of skin diseases in our environment would aid the health-care provider to appropriately plan and execute policies that would help to assist and ameliorate the suffering of our people, in this era of HIV/AIDS pandemic that is gradually changing the face of tropical dermatology in our time. We report our findings and compare it with other zones in Nigeria and some parts of Africa and the world.

## 2. Materials and Methods

### 2.1 Methods

This was a 12month prospective study of all new patients seen in Dermatology/Venereology out-patients clinic of University of Benin Teaching Hospital, Benin City, Edo State, South-South geo-political zone of Nigeria between September, 2006 and August, 2007. Data were compared with findings from other geo-political zones in Nigeria and worldwide.

### 2.2 Procedures

All patients were seen by the researchers. The patients’ age, sex and skin disease diagnosis were made clinically and histopathologically where necessary and documented. Fungal studies which included skin scraping for potassium hydroxide microscopy and cultured in sabourauds medium were done. Bacterial cultures were performed, skin snip for microfilaria and slit-skin smear for acid and alcohol fast bacilli of mycobacterium lepral were done. Few patients that had connective tissue disease benefitted from immunological studies and HIV tests were also done. Patch test was not done.

### 2.3 Statistical Analysis

The data collected were categorized and the frequencies of the main disease diagnosis determined and tabulated according to the frequencies of their presentation and percentages calculated. The tables and figure was done using Microsoft Excel 2007.

## 3. Results

### 3.1 Analysis

Within the 12 months of the study period, a total number of 4786 patients comprising 2674 HIV/AIDS patients and 2112 pure dermatological cases were seen at the dermatology/Venereology out-patient clinic. Out of this number, 755 were new patients. Majority of the patients were from Benin and the others were from neighbouring towns and states either on self-referrals or hospital referrals.

There were 354 males (46.9%) and 401 females (53.1%), with a female/male ratio of 1.1:1. The ages of the patients ranged from 2 weeks to 15years for the children and 16 years to 80 years for the adults. About fifty-five (55) (7.3%) had more than one dermatological diagnosis.

**Figure 1:**
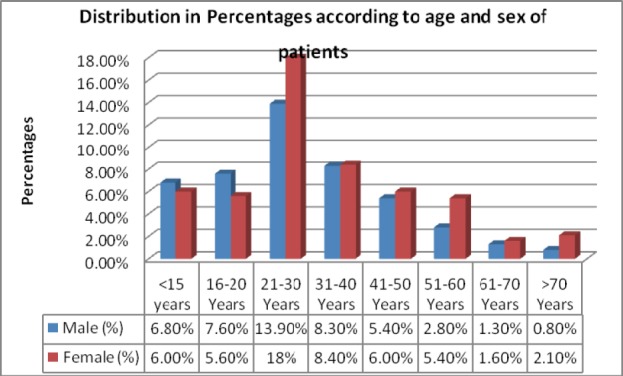
shows the percentages of Age and Sex Distribution of the patients that had skin diseases

Table 1 shows the frequencies of the main disease groups of skin diseases as seen in Benin City.

**Table 1:** Main disease groups of skin diseases

Skin Disease	Frequency	%
Eczematous Dermatitis	158	20.9
Papulosquamous Disorders	68	9
Fungal Infections	60	7.9
Human Immunodeficiency virus- related skin disease	60	7.9
Viral Infections	58	7.6
Benign Neoplastic Skin Disorders	49	6.5
Pigmentary Disorders	38	5
Pilosebaceous Disorders	38	5
Urticaria/Urticaria- like Disorders	36	4.7
Hair Disorders	31	4.1
Adverse Drug Eruptions	30	4.1
Disorders of Cornifications	20	2.6
Bacterial Infections	18	2.5
Parasitic Infections	16	2.2
Genodermatitis	15	2
Pruritis	14	1.9
Connective Tissue Disorder	13	1.7
Cutaneous Malignancies	7	1
Lymphedema	6	0.8
Miscellaneous Skin Disease	20	2.6
**Total**	**755**	**100**

Table 2 shows the breakdown of the various categories of skin diseases seen. This table reveals that eczematous dermatitis is the commonest skin disease prevalent in our environment (20.9%). Contact and atopic dermatitis were 35% and 29% respectively in their group and this seems to be the observable pattern throughout the country. Infectious/infestations accounted for 20.1% when combined and this is important when we consider our environment and the available social infrastructure that is rapidly deteriorating. Papulosqamous and pigmentation disorder (9%) and (5%) respectively are also observed to be on the increase.

**Table 2:** Various categories of skin diseases

SKIN DISEASES	FREQUENCY	%	%
**Eczematous Dermatitis:**			
Contact Dermatitis	56	35.4	7.4
Atopic Dermatitis	46	29.1	6.1
Photo Dermatitis	8	5.1	1.1
Seborreheic Dermatitis	6	3.8	0.8
Exfoliative Dermatitis	6	3.8	0.8
Stasis Dermatitis	4	2.5	0.5
Pomphylox	4	2.5	0.5
Lichen Simplex Chronicus	4	2.5	0.5
Non Specific Chronic Dermatitis	24	15.3	3.2
**Group Total**	**158**	**100**	**20.9**

**Papulosquamous Disorder:**			
Lichen Planus	24	35.3	3.2
Pityriasis Rosea	24	35.3	3.2
Psoriasis	20	29.4	2.6
**Group Total**	68	100	9

**Urticaria and other Pruritic Disorder:**			
Urticaria	18	50	2.4
Papular Urticaria	12	33.3	1.5
Physciar Urticaria	4	11.1	0.5
Others	2	5.6	0.3
**Group Total**	**36**	**100**	**4.7**

**Pilosebacious Disorder:**			
Acne Vulgaris	34	89.5	4.5
Other Acneiform Disorder	4	10.5	0.5
**Group Total**	**38**	**100**	**5**

**Adverse Drug Eruptions:**			
Fixed Drug Eruptions	14	46.6	1.8
Lichenoid Drug Eruptions	4	13.2	0.5
Exanthema	2	6.7	0.3
Acneiform Drug Eruptions	2	6.7	0.3
Angioneurotic Edema	2	6.7	0.3
Steroid Induced Striae	2	6.7	0.3
Steven Johnson’s Syndrome	2	6.7	0.3
Urticaria Drug Eruptions	2	6.7	0.3
**Group Total**	**30**	**100**	**4.1**

**Viral Infections:**			
Viral Warts	34	58.6	4.5
Herpes Zoster	14	24.2	1.8
Herpes Simplex Virus	6	10.3	0.8
Molluscum Contagiosum	4	6.9	0.5
**Group Total**	**58**	**100**	**7.6**

**Bacteria Infections:**			
Hansen’s Disease	5	27.8	0.6
Cellulitis	3	16.7	0.4
Impetigo	2	11.1	0.3
Furunculosis	2	11.1	0.3
Ecthyma	2	11.1	0.3
Folliculitis	2	11.1	0.3
Others	2	11.1	0.3
**Group Total**	**18**	**100**	**2.5**

**Fungal Infections:**			
Dermatophyte Infections	24	40	3.2
Pityriasis Vesicolor	18	30	2.4
Candidiasis	4	6.7	0.5
Other Fungal Infections	14	23.3	1.8
**Group Total**	**60**	**100**	**7.9**

**Parasitic Infestations:**			
Scabies	8	50	1.1
Onchocerciasis	6	37.5	0.8
Tungiasis	2	12.5	0.3
**Group Total**	**16**	**100**	**2.2**

**Pigmentary Disorders:**			
Vitiligo	24	63.1	3.2
Albinism	4	10.5	0.5
Idiopathic Guttate Hypomelanosis	2	5.3	0.3
Piebaldism	2	5.3	0.3
Post Inflammatory Hypopigmentation	2	5.3	0.3
Other Disorders Of Pigmentation	4	10.5	0.5
**Group Total**	**38**	**100**	**5.1**

**GenoDermatoses:**			
Neurofibromatosis 1	14	93.3	1.8
Plexiform Neurofibromatosis	1	6.7	0.1
**Group Total**	**15**	**100**	**1.9**

**Cutaneous Malignancies:**			
Endemic Kaposi’s Sarcoma	2	28.6	0.3
Basal Cell Carcinoma	2	28.6	0.3
Squamous Cell Carcinoma	2	28.6	0.3
Melanoma	1	14.2	0.1
**Group total**	**7**	**100**	**1**

**Pruritus:**			
Generalized Pruritus	12	85.7	1.5
Psychogenic Pruritus	2	14.3	0.3
**Group Total**	**14**	**100**	**1.8**

**HIV-Related Skin Diseases:**			
Kaposi’s Sarcoma	14	23.3	1.8
Pruritic Papular Eruptions of HIV	10	16.7	1.3
Drug Eruptions	10	16.7	1.3
Herpes Zoster	8	13.4	1.1
Dermatophyte Infections	8	13.4	1.1
Molluscum Contagiosum	2	3.3	0.3
Squamous Cell Carcinoma of the penile shaft	1	1.6	0.1
Psoriasis	1	1.6	0.1
others	6	10	0.8
**Group Total**	**60**	**100**	**7.9**

**Hair Disorders:**			
Acne keloidalis Nuchea	12	38.7	1.5
Pseudofolliculitis Barbae	8	25.8	1.1
Alopecia Areata	6	19.3	0.8
Folliculitis Decalvans	2	6.5	0.3
Trichotillomania	1	3.2	0.1
Others	2	6.5	0.3
**Group Total**	**31**	**100**	**4.1**

**Connective Tissue Diseases:**			
Systemic Lupus Erythematosis	6	46.1	0.8
Systemic Sclerosis	2	15.4	0.3
Discoid lupus Erythematosis	2	15.4	0.3
Others	3	23.1	0.4
**Group Total**	**13**	**100**	**1.8**

**Disorder of Cornification:**			
Follicular Hyperkeratosis	8	40	1.1
Tylosis	4	20	0.5
Hereditary Icthyosis	2	10	0.3
Pharynoederma	2	10	0.3
Others	4	20	0.5
**Group Total**	**20**	**100**	**2.7**

**Lymphoedema:**			
Acquired Lymphoedema	4	66.7	0.5
Congenital Lymphoedema	2	33.3	0.3
**Group Total**	**6**	**100**	**0.8**

**Benign Neoplastic Skin Disease:**			
Adenoma Sebaceum	8	50	1.1
Syringoma	3	18.7	0.4
Dermatosis Papulosa Nigra	2	12.6	0.3
Others	3	18.7	0.4
**Group** **Total**	**16**	**100**	**2.2**

**Fibrous Tumours of Skin:**			
Keloids	14	42.4	1.8
Epidermal Nevus	10	30.3	1.3
Actinic Keratosis	4	12.1	0.5
Hypertrophic Scars	2	6.1	0.3
Others	3	9.1	0.4
**Group Total**	**33**	**100**	**4.3**

**Miscellaneous Skin Disorders**	20		2.5

Table 3 shows the comparison of incidence (%) of common dermatoses as observed from different zones in Nigeria and other parts of Africa and the world. Table 4: shows miscellaneous skin disorders.

**Table 3:** Comparison of incidence in percentages (%) of common dermatoses as observed from different zones in Nigeria and other parts of Africa and the world

Skin Diseases	This study 2006-2007 Benin n=755	Ogunbiyi et al. 1994-1998 Ibadan n=1091	Nnoruka et al. 1999-2001 Enugu n=2871	Husain et al. 2000-2005 Kaduna n=5982	Souissi et al. 1999-2000 Tunis n=28,294	Hartshorne et al. 1999- Johannesburg n=5335	Mitchell et.al Canada
	%	%	%	%	%	%	%
Contact Dermatitis	7.4	0.7	5.3	5.8	-	18.8	
Atopic Dermatitis	6.1	5.8	4.8	13.8	0.72	-	39.2
Non Specific Chronic Disease	3.2	-	-	-	-	-	-
Acne Vulgaris	4.5	2.8	4.3	6.7	6.9	-	-
Viral Warts	4.5	2	0.7	2.9	-	3.7	7.3
Lichen Planus	3.2	3.4	4.8	1.2	-	1	6.8
Pityriasis Rosea	3.2	1.6	4.1	2.1	11.1	0.9	-
Dermatophyte Infections	3.2	4.5	8.3	6	-	-	-
Vitiligo	3.2	5.7	3.2	2	3.4	1.3	4.7
Psoriasis	2.6	0.9	0.5	1.2	1.9	2.1	-
Urticaria	2.4	4.6	2.3	3.6	3.11	1.9	-
Pityriasis vesicolor	2.4	4.5	1.7	2.4	-	-	-
Fixed Drug Eruptions	1.8	2.2	1.1	2.7	-	1.1	-
Herpes Zoster	1.8	0.6	0.7	0.8	0.22	3.2	-
Neurofibromatosis	1.8	0.9	0.7	0.2	-	-	-
Kaposi’s sarcoma (epidemic)	1.8	-	-	0.5	-	-	-
Keloids	1.8	-	-	-	-	-	-
Papular Urticaria	1.5	1.1	1.3	3.6	-	-	-
Generalized Pruritis	1.5	-	-	-	-	-	-
Acne Keloidilis Nuchea	1.5	2.9	3.7	0.5	-	0.5	-
Drug Eruptions in HIV/AIDS	1.3	-	-	-	-	-	-
Papular Pruritic Dermatosis in HIV/AIDS	1.3	-	1.4	1.8	-	0.4	-

**Table 4:** Miscellaneous skin diseases

Miscellaneous Skin Disorders:	Frequency
Sexually Transmitted Diseases	2
Miliaria	2
Striae Distensae	2
Pyoderma Gangrenosum	1
Cutaneous Leishmaniasis	1
Aplasia cutis	1
Prurigo Nodularis	1
Chronic Leg Ulcer	3
Venereophobia	1
Post Herpetic Neuralgia	3
Lipoma	1
Recurrent Oral Aphthous Ulcer	1
Acanthosis Nigricans	1

**Total**	20

New emerging disease entities due to HIV accountable for 7.9% and epidemic Kaposi’s sarcoma is also on the increase, involving younger age group as compared to classical Kaposi’s sarcoma. Connective tissue disorder and cutaneous malignancies were less commonly observed (1.7% and 1.1% respectively). Neurofibromatosis was the only genodermatosis observed during the study period and it accounted for 1.9% of the skin diseases and interestingly all were females. The hair disorder accounted for 4.1% of the skin diseases and acne keloidalis nuchea topped the list with 38.7% in their group and this was mostly observed among young adults who have been exposed to one form of hairdo or the other.

## 4. Discussion

This study describes the pattern of skin diseases in Benin City, Edo State, south-south geo-political zone of Nigeria. The University of Benin Teaching Hospital is a tertiary hospital which receives referrals from within and neighbouring states. Benin is a cosmopolitan city in the South- South region of Nigeria and they are known for their ancient traditional and cultural beliefs. It has approximately 1.3 million inhabitants and the city is located in Edo state southern Nigeria (longitude 5.6145 ◻ E and latitude 6.3176 ◻ N). The average daily temperature ranges from 28- 37 ◻ C throughout the year with low humidity most times and regular rainfall.

We discovered that eczematous dermatitis was the commonest dermatoses and this finding was similar to studies done elsewhere in other regions of Nigeria like the south-east, south-west and north- central of Nigeria respectively (Nnoruka, 2005; Ogunbiyi, Daramola & Alese, 2004; Husain, 2007). This is in contrast to initial findings decades ago were infections/infestation was the commonest skin disease (Husain, 2007; Fekete, 1978; Onayemi, Isezuo, & Njoku, 2005; Schemeller & Dzikus, 2001) in most regions of the country, and other parts of Africa. The rapid industrialization in most major cities in Nigeria and the attending overcrowding in search of well paid jobs in the cities may probably have accounted for this observation. The high occurrence of eczematous dermatitis with contact dermatitis topping the list (35.4%) in their group, might be attributed to the high density of Petro-chemical industries and other irritants like rubber, dyes, cosmetics, etc. within the Niger-Delta region where Benin city is located. The above observation about the increasing prevalence of contact dermatitis is important in the sense that it would help to create awareness among patients, attending community physician and also alert the government on the need for proper planning and adequate compensation to those who might have had the disease as a result of occupational hazard. The incidence of infections/infestations skin diseases might have been overtaken by eczematous dermatitis but its high percentage occurrence is still a thing of worry. To sustain the gain of reduction in prevalence of infectious skin diseases, government need to maintain their bit by providing more good houses, clean drinking/bathing water and availability of drugs such as anti-microbials (Onayemi, Isezuo, & Njoku, 2005).

The changing face of most dermatological diseases among patients having sexually transmitted infections and HIV/AIDS was also noticed in this study. Epidemic Kaposi’s sarcoma which was hitherto considered to be rare accounted for majority of the presentation and this was contrary to previous findings: in Enugu (Nnoruka, 2005) where seborrheoic dermatitis was the most prevalent; Kaduna (Husain, 2007) where papular pruritic dermatosis was most prevalent; or in Daressalamm, Tanzania (Gibbs, 1996) where herpes zoster was the most prevalent. The reason for this observation cannot readily be adduced, however, host factors and environmental factors in a prevailing depressed immunity might have accounted for that. It has been observed that people living in the Mediterranean region of this world, East Africa, and those on cytotoxic drugs are prone to having classical, endemic and sporadic Kaposi’s sarcoma respectively (Centers For Disease Controls, 1982).

Papulosquamous skin disorders were also observed to be on the increase. Although it is rarely life threatening, its effect on quality of life can be devastating to the patients. In fact, the marriage of one of the women that had generalized chronic plaque psoriasis was threatened if she was not cured. Acne vulgaris was most prevalent Pilosebaceous disorders, and this was similar to the findings in the other parts of the country (Nnoruka 2005; Ogunbiyi, Daramola, & Alese 2004; Husain 2007; Stathakis, Kilkenny, & Marks, 1997). Most of these patients were secondary school students and university undergraduates. Among this age group, there is conscious awareness of their look and self image before their peers (Onayemi, Isezuo, & Njoku, 2005; Chan & Rohr, 2000).

Pigmentary disorders were observed to be on the increase. Vitiligo and albinism were the most prevalent. Vitiligo cases were on the increase possibly due to its comestic impact on the patients and fear of more sinister underlying diseases (Nnoruka, 2005; Onunu & Kubeyinje, 2003). Most of the cutaneous malignancies observed in this study were on albinos which suggest that a lot needs to be done in creating awareness on the avoidance of ultra-violet rays which has been highly implicated (Okoro, 2006). The only genodermatosis observed in this study was neurofibromatosis type 1/2 which accounted for 1.9% of the skin diseases. This is on the increase when compared with the figures obtained in Enugu, Ibadan and Kaduna (Nnoruka, 2005, Ogunbiyi, Daramola, & Alese, 2004; Husain, 2007). Reasons for this might just have been a chance occurrence.

## 5. Conclusion

In conclusion, our findings showed no major differences in the prevalence of skin diseases published in previous literatures from other geo-political zones of the country and Africa. The high incidence of eczematous dermatitis observed in this study might be attributed to on-going industrialization and rural- urban migration. Sexually transmitted infections and HIV/AIDS related skin diseases were on the increase and might in the future change the manifestations of most cutaneous skin diseases in tropical dermatology. Health care providers need to be properly informed about these changes and well articulated plans policies should be put in place for implementation.
